# Supplementary material for: From sequence to enzyme mechanism using multi-label machine learning
Source: BMC Bioinformatics. 2014 May 19;15:150. doi: 10.1186/1471-2105-15-150 (PMC4229970; doi:10.1186/1471-2105-15-150)
Supplement: Additional file 2 — Java code of ml2db. Additional file ml2db_code.tar.gz contains the Java source code to run the multi-label machine learning experiments and save the results to database. The code’s Javadoc is included. [file 1471-2105-15-150-S2.zip › additional file 2/ml2db/ml2db/doc/main/ml2db/graphviz/GraphVizArffColoured.html]

GraphVizArffColoured


JavaScript is disabled on your browser.


- Overview
- Package
- Class
- Use
- Tree
- Deprecated
- Index
- Help

- Prev Class
- Next Class

- Frames
- No Frames

- All Classes

- Summary:
- Nested |
- Field |
- Constr |
- Method

- Detail:
- Field |
- Constr |
- Method


main.ml2db.graphviz

## Class GraphVizArffColoured

- java.lang.Object
- - main.ml2db.graphviz.GraphViz
  - - main.ml2db.graphviz.GraphVizArff
    - - main.ml2db.graphviz.GraphVizArffColoured

- ---

    

  ```
  public class GraphVizArffColoured
  extends GraphVizArff
  ```

  Nodes and edges can of a base dot file can be coloured to show their labelled
  status (train set), or their correctly/uncorrectly predicted status (test
  set).

  Version:
  :   15 Apr 2012

  Author:
  :   Luna De Ferrari ldf(at)st-andrews.ac.uk or
      luna.deferrari@st-andrews.ac.uk

- - ### Field Summary

    Fields

    | Modifier and Type | Field and Description |
    | --- | --- |
    | `static java.lang.String` | `LABELLED_ATTRIBUTE_SHAPE` |
    | `static java.lang.String` | `LABELLED_CLASS_SHAPE` |
    | `static java.lang.String` | `LABELLED_INSTANCE_SHAPE` |

    - ### Fields inherited from class main.ml2db.graphviz.GraphVizArff

      `BASE_ATTRIBUTE_SHAPE, BASE_CLASS_SHAPE, BASE_INSTANCE_SHAPE`
    - ### Fields inherited from class main.ml2db.graphviz.GraphViz

      `DOT, DOT_COMMENT_PREFIX, DOT_UNDIRECTED_EDGE, FIG, GIF, JPG, PDF, PLAIN, PNG, PS, SVG`
  - ### Constructor Summary

    Constructors

    Constructor and Description

    `GraphVizArffColoured(java.lang.String title)`
  - ### Method Summary

    Methods

    | Modifier and Type | Method and Description |
    | --- | --- |
    | `void` | `colourLabelled(IdMultiLabelInstances labelledInstances)` Assigns a colour depending on an integer (from 0 to >4) |
    | `void` | `colourPredictedInstances(java.util.TreeMap<IdInstance,java.lang.Double> instanceToPercentageMap)` |

    - ### Methods inherited from class main.ml2db.graphviz.GraphVizArff

      `addInstances, completeDotLine, fillDot, getBaseDotAllInstances`
    - ### Methods inherited from class main.ml2db.graphviz.GraphViz

      `add, addln, addln, end_graph, getDotSource, getGraphImage, readSource, start_graph, toGif, toGifToTmp, toImage, toImageFile, toJpg, toPdf, writeDotSourceToFile, writeGraphToFile, writeGraphToFile`
    - ### Methods inherited from class java.lang.Object

      `equals, getClass, hashCode, notify, notifyAll, toString, wait, wait, wait`

- - ### Field Detail


    - #### LABELLED\_INSTANCE\_SHAPE

      ```
      public static final java.lang.String LABELLED_INSTANCE_SHAPE
      ```

      See Also:
      :   Constant Field Values


    - #### LABELLED\_CLASS\_SHAPE

      ```
      public static final java.lang.String LABELLED_CLASS_SHAPE
      ```

      See Also:
      :   Constant Field Values


    - #### LABELLED\_ATTRIBUTE\_SHAPE

      ```
      public static final java.lang.String LABELLED_ATTRIBUTE_SHAPE
      ```

      See Also:
      :   Constant Field Values
  - ### Constructor Detail


    - #### GraphVizArffColoured

      ```
      public GraphVizArffColoured(java.lang.String title)
      ```

      Parameters:
      :   `instances` -
  - ### Method Detail


    - #### colourLabelled

      ```
      public void colourLabelled(IdMultiLabelInstances labelledInstances)
      ```

      Assigns a colour depending on an integer (from 0 to >4)

      Parameters:
      :   `identifier` -
      :   `number` - an integer (only the first 4 levels are coloured)
          private void colourByNumber(String identifier, int number) {
          // example of series from "good" to "bad" (from 0% to 100%):
          // green3/green, lawngreen/greenyellow/yellow, orange, red
          if (number == 1) { this.addln(identifier +
          this.getStyle("red")); } else if (percentage >= 0.50 &&
          percentage < 0.75) { this.addln(identifier +
          this.getStyle("orange")); } else if (percentage >= 0.75 &&
          percentage < 1.0) { this.addln(identifier +
          this.getStyle("yellow")); } else if (percentage == 1.0) {
          this.addln(identifier + this.getStyle("green3")); }
          }


    - #### colourPredictedInstances

      ```
      public void colourPredictedInstances(java.util.TreeMap<IdInstance,java.lang.Double> instanceToPercentageMap)
      ```


- Overview
- Package
- Class
- Use
- Tree
- Deprecated
- Index
- Help

- Prev Class
- Next Class

- Frames
- No Frames

- All Classes

- Summary:
- Nested |
- Field |
- Constr |
- Method

- Detail:
- Field |
- Constr |
- Method
